# Supplementary material for: QTLs Related to Rice Callus Regeneration Ability: Localization and Effect Verification of qPRR3
Source: Cells. 2022 Dec 19;11(24):4125. doi: 10.3390/cells11244125 (PMC9777078; doi:10.3390/cells11244125)
Supplement: Supplementary file 1 [file cells-11-04125-s001.zip › cells-2112472-supplementary.pdf]

Table S1 Available InDel markers screened in *qPRR3*

InDel	InDel loc	InDel size	9311 (—)	182	Nip (+)	Promter			
						F/R	loc	TM	sequence
InDel 3-1	2756129	34	141	+	175	F	2756090	60	AAAGGGACCCTAAAAGCGCC
						R	2756265	60	CGTTTTATCCGGGGCGGCTA
InDel 3-2	2799657	24	247	+	271	F	2799524	57	GCTGTTAACTGGTACTCCGTT
						R	2799795	57	GCTAGCTAGCTAGTAGCATGG
InDel 3-3	2811484	17	198	+	181	F	2811447	56	CGTTCCAATTTCAACCGAACTTTT
						R	2811628	58	TCAGATTCGTACGCCGTCAT
InDel 3-4	2913200	41	206	+	165	F	2913158	54	GTGTTGTGACAAAATCAATGATACC
						R	2913323	58	TACGGATTTATTGCCACCACCA
InDel 3-5	2983799	31	225	—	256	F	2983724	58	CACATGATCCTACTCAAAAAAG
						R	2983980	57	CCATCCTACTAAAGTATCATATC

Five available InDel markers were screened in *qPRR3*.

Table S2 The relative expression levels of all genes in *qPRR3* in parent and RILs

Gene ID	CDS Coordinates (5'-3')	relative expression			Gene Product Name
		Nip	93-11	BC_3F_2-182-78	
LOC_Os03g05540	2757582 - 2762244	12.10	15.06	14.42	tetratricopeptide repeat containing protein, putative, expressed
LOC_Os03g05550	2767839 - 2768531	0.37	0.00	0.24	expressed protein
LOC_Os03g05560	2772434 - 2771187	0.06	0.05	0.03	zinc finger, C3HC4 type domain containing protein, expressed

LOC_Os03g05570	2777769 - 2776612	0.56	0.39	0.89	RING-H2 finger protein ATL3F, putative, expressed
LOC_Os03g05580	2793544 - 2794224	0.00	0.00	0.01	expressed protein
LOC_Os03g05590	2800290 - 2800924	0.13	0.05	0.00	AP2 domain containing protein, expressed
LOC_Os03g05600	2802871 - 2803283	0.00	0.00	0.00	hypothetical protein
LOC_Os03g05610	2804141 - 2805943	0.13	0.01	0.06	inorganic phosphate transporter, putative, expressed
LOC_Os03g05620	2809678 - 2807555	18.12	22.22	25.70	inorganic phosphate transporter, putative, expressed
LOC_Os03g05630	2811738 - 2814268	1.03	1.39	1.47	expressed protein
LOC_Os03g05640	2817367 - 2815438	0.43	1.00	0.01	inorganic phosphate transporter, putative, expressed
LOC_Os03g05650	2818997 - 2820125	0.05	0.01	0.03	expressed protein
LOC_Os03g05660	2828096 - 2822929	5.67	15.55	8.63	appr-1-p processing enzyme family protein, putative, expressed
LOC_Os03g05680	2833133 - 2837273	15.88	11.21	17.82	histone demethylase JARID1C, putative, expressed
LOC_Os03g05690	2838772 - 2841691	33.54	20.23	28.19	ZOS3-03 - C2H2 zinc finger protein, expressed
LOC_Os03g05700	2842391 - 2841790	20.25	0.80	13.78	expressed protein
LOC_Os03g05710	2844831 - 2843447	9.11	6.66	14.57	acetyltransferase, GNAT family, putative, expressed
LOC_Os03g05720	2845709 - 2851689	44.64	44.02	45.36	WD domain, G-beta repeat domain containing protein, expressed
LOC_Os03g05730	2856682 - 2852293	102.40	122.74	97.05	cell division control protein 48 homolog E, putative, expressed
LOC_Os03g05740	2860232 - 2857125	11.95	11.64	10.97	ras-related protein, putative, expressed
LOC_Os03g05750	2868242 - 2866315	22.15	8.74	22.87	heavy-metal-associated domain-containing protein, putative, expressed
LOC_Os03g05760	2870362 - 2875430	9.86	7.06	4.94	transcription factor Dp, putative, expressed
LOC_Os03g05770	2878828 - 2880890	0.07	0.12	0.15	peroxidase precursor, putative, expressed
LOC_Os03g05780	2887922 - 2883005	15.64	13.26	18.57	4-coumarate--CoA ligase-like 7, putative, expressed
LOC_Os03g05800	2900285 - 2897476	1.23	1.75	1.49	expressed protein
LOC_Os03g05806	2901596 - 2906614	3.78	3.29	3.58	pseudouridine synthase family protein, putative, expressed
LOC_Os03g05812	2907108 - 2912670	15.52	18.48	15.45	expressed protein

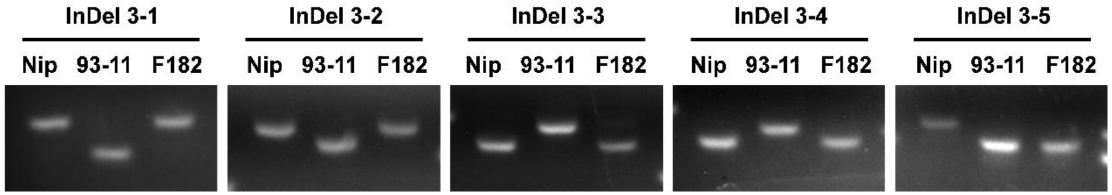

Figure S1 InDel marker of *qPRR3*

Five available InDel markers in *qPRR3*. InDel 3-1 to InDel 3-4, F182 showed the genotype of Nip. InDel 3-5, F182 showed the genotype of 93-11.
